# Development of a New Embedded Dynamometer for the Measurement of Forces and Torques at the Ski-Binding Interface

**DOI:** 10.3390/s19194324

**Published:** 2019-10-07

**Authors:** Frédéric Meyer, Alain Prenleloup, Alain Schorderet

**Affiliations:** 1 Sport Science Institute, University of Lausanne, CH-1015 Lausanne, Switzerland; 2 Ecole Polytechnique Fédérale de Lausanne (EPFL), School of Engineering (STI), Mechanical Engineering Section, Station No. 9, CH-1015 Lausanne, Switzerland; alain.prenleloup@epfl.ch; 3 University of Applied Sciences and Arts Western Switzerland (HES-SO), HEIG-VD, CH-1401 Yverdon-les-Bains, Switzerland; alain.schorderet@heig-vd.ch

**Keywords:** alpine skiing, force platform, giant slalom, elite athletes, field testing

## Abstract

In alpine skiing, understanding the interaction between skiers and snow is of primary importance for both injury prevention as well as performance analysis. Risk of injuries is directly linked to constraints undergone by the skier. A force platform placed as an interface between the ski and the skier should allow a better understanding of these constraints to be obtained to thereby develop a more reliable release system of binding. It should also provide useful information to allow for better physical condition training of athletes and non-professional skiers to reduce the risk of injury. Force and torque measurements also allow for a better understanding of the skiers’ technique (i.e., load evolution during turns, force distribution between left and right leg…). Therefore, the aim of this project was to develop a new embedded force platform that could be placed between the ski boot and the binding. First, the physical specifications of the dynamometer are listed as well as the measurement scope. Then, several iterations were performed on parametric 3D modeling and finite element analysis to obtain an optimal design. Two platforms were then machined and equipped with strain gauges. Finally, the calibration was performed on a dedicated test bench. The accuracy of the system was between 1.3 and 12.8% of the applied load. These results show a very good linearity of the system, which indicate a great outcome of the design. Field tests also highlighted the ease of use and reliability. This new dynamometer will allow skiers to wear their own equipment while measuring force and torque in real skiing conditions.

## 1. Introduction

In alpine skiing, force platforms were first developed to understand the mechanism of knee injuries to try to find solutions to improving the safety of the bindings. Hull and Mote [1,2,3] proposed a system consisting of two independent six degrees of freedom dynamometers integrated in the ski, below the bindings. Another design was proposed by MacGregor et al. [4], who aimed to develop an electronic released binding system to record data. The binding was integrated between the ski and the boot and the release algorithm was discussed in another article [5]. A second generation of devices was presented by Wunderly et al. [6], who dedicated special attention to maximizing the mechanical decoupling of the load and reducing the cross sensitivity between components. Nevertheless, the accuracy of the system was not clearly defined. A revised design of Hull’s first force platform [1] was also proposed by Quinn and Mote [7] by using instrumented T-shaped shear panel elements. Aiming to predict constraints undergone by the knee during skiing to prevent injuries, Quinn and Mote [8] used their system, in addition to a goniometer to measure the ankle, to determine the forces and moments at the boot top and the knee. Concerned with the possible effect of the bending of the skis on the measure of vertical load, Wimmer and Holzner [9] developed two different devices measuring the vertical reaction force. The first was inserted between the skis and the binding, and the second between the binding and the boot. The first design was significantly perturbed by the bending of the skis, but not the second.

More recently, new systems have been developed to allow for force and moment measurements on both skis, necessary for a complete understanding of the kinetics. Vodickova et al. [10] proposed a device based on strain gauges that substituted the plate on the carving ski, raising the skier by 6 mm compared to the usual position. Several studies involving a force platform developed by Kistler (Kistler AG, Winterthour, Switzerland) based on piezoelectric sensors, have been published since 2000 [11,12,13]. In her study of carving turns, Klous [13] found maximal vertical loads around 2.5 N/kg (of body mass) on the outer leg. Medio-lateral and anterio-posterior forces were equally distributed between the external and internal legs, but a lot smaller than the vertical forces (approximately 1 N/kg for both components). Fore-aft torques were measured between −2 and 2 Nm/kg for both legs. Maximum measured abduction–adduction moments were around 0.5 Nm/kg and internal–external rotations were approximately 0.3 Nm/kg on the outside leg. The detailed protocol concerning the Kistler plate validation was published by Stricker et al. [14]. The achieved sampling rate could reach up to 500 Hz, each dynamometer was 3.2 cm high, and weighed 1.8 kg. The cross talk between the components ranged between 0.2 and 3.6%, depending on the axis. To control the drift induced by piezoelectric sensors, the dynamometers had to be zeroed at the beginning and the end of the measurement. The results showed a very low influence of the temperature (between 0.3 and 1.73%), and an average increase in the relative accuracy with an increase in the vertical load (4.6–0.3%). The accuracy in the other directions was lower than 2.5%, while torque accuracy was ranked between 4.0 and 8.3%. The persisting issue of all of the proposed solutions is the need to modify the skiing equipment, and therefore the impossibility for the skier to use their own material.

Kiefmann et al [15] developed an interesting force platform, as the device could be fixed as an interface between the ski boot and the binding without any modification of the system. Unfortunately, the accuracy of the system was not specified and the platform suffered from mechanical weakness. A mock-up of the platform, with similar dimensions (i.e., 4 cm high and 2 kg each) was used to determine the influence of the material during moguls skiing [16]. No significant differences were found in the kinematic parameters (i.e., knees angles, forward lean of the torso, hips forward and lateral inclinations) when using the devices. 

There is a need for fully integrated force platforms that will allow skiers to ride with their own equipment. The aim of this project was therefore to design a compact dynamometer with dimensions so that it could be placed as a removable interface inserted between the boot and the binding. 

## 2. Materials and Methods

### 2.1. Dynamometer Design

The force platform was designed to fit a ski boot length of 310 mm to 315 mm, with an International Organization for Standardization (*ISO*) compatible interface to fit in the standard ski bindings. The total height needed to be less than 25 mm. Nominal loads were inspired from the literature review as well as from theoretical models. The dynamometer was planned to measure maximal vertical forces (*F_Z_*) of 3000 N, lateral forces (*F_Y_*) of 1000 N, torques around the Y axis (*M_Y_*) of 500 Nm, around the Z axis (*M_Z_*) of 100 Nm, and around the X axis (*M_X_*) of 150 Nm. Frontal forces (*F_X_*) were not considered in this development, as forces (e.g., ski-snow friction) are very low in this direction.

**Figure 1:**
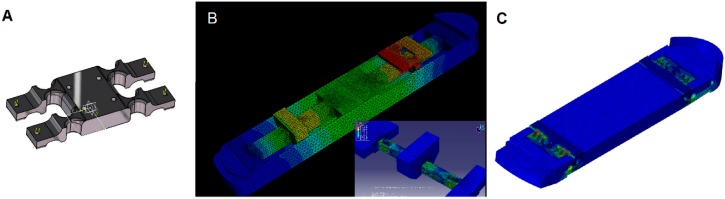
(**A**) First iteration of the design. (**B**) Second iteration of the design with two sensors fixed to a rigid bed. (**C**) Final iteration with an integrated bed and ski binding interface. Sensors were placed on the front and back of the plate. Colors represent the von Mises values (blue: no deformation; red: maximal deformation).

**Figure 2:**
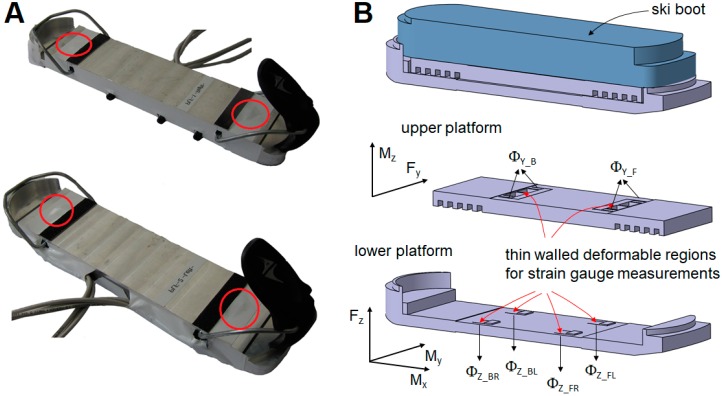
(**A**) The two manufactured forces platforms with sensors position marked in red. (**B**) Schematic view of the sensor, composed of two stages. The upper part allows the *Fy* and *Mz* components to be measured, while the lower part aims to measure *Fz*, *Mx*, and *My*. *Φ* indicates the different recorded signals.

A fully integrated solution was chosen to fulfil the specifications. In addition to the latter, the sensor’s bandwidth was defined as 0–20 Hz, since the frequency of human movements never exceeds 20 Hz [17]. To meet this requirement, the minimum sensor’s stiffness values were defined to provide skier–sensor eigen-frequencies above 100 Hz. The most critical modes considered were the ones involving the binding–skier as an inertia and the ski as a compliance. Then, using the dynamic displacement amplification factor of the harmonic loading *μ*, the bandwidth can be computed using the following equation:

(1) $$\mu \left(f\right)=\frac{s_{d}}{s_{s}}=\frac{1}{\sqrt{{\left(1-{\left(\frac{f}{f_{r}}\right)}^{2}\right)}^{2}+4\eta_{r}^{2}{\left(\frac{f}{f_{r}}\right)}^{2}}}$$

where μ is the amplification factor; s_d_ the dynamic signal; s_s_ the corresponding static signal (response if the excitation was static, i.e., at *f* = 0); f is the excitation frequency; f_r_ the r-th Eigen frequency; and η_r_ is the corresponding modal damping. We considered the worst case as η_r_ = 0. Applying *f* = 20 Hz and *f_r_* = 100 Hz in the above formula ensures that the measurement errors caused by the sensor’s eigen modes are below 4.1% for each signal up to 20 Hz. Therefore, as a first step, a static calibration of the dynamometer will allow the dynamic measurement of the different components within these specifications. Several iterations were performed on parametric 3D modeling and finite element analysis to obtain an optimal design (Figure 1A–C). Two force platforms were then machined (Figure 2A), and strain gauges were layered on the dedicated flexible elements in a half Wheatstone bridge configuration, decoupling lateral and vertical loads. Figure 2B shows a schematic representation of the dynamometer with the different measured elements. Vertical loads were measured using four strain gauge signals of the lower platform: *Φ_Z_FL_* (front left), *Φ_Z_FR_* (front right), *Φ_Z_BL_* (back left), and *Φ_Z_BR_* (back right). These signals were combined as defined in Equations (2) and (3) to measure the vertical load F_Z_ and moment *M_X_*.

(2) $$F_{Z}=\left(C_{ZF\_FL}+C_{ZB\_FL}\right)\Phi_{Z\_FL}+\left(C_{ZF\_FR}+C_{ZB\_FR}\right)\Phi_{Z\_FR}+\left(C_{ZF\_BL}+C_{ZB\_BL}\right)\Phi_{Z\_BL}+\left(C_{ZF\_BR}+C_{ZB\_BR}\right)\Phi_{Z\_BR}$$

(3) $$M_{X}=\left(C_{mXF\_FL}+C_{mXB\_FL}\right)\Phi_{Z\_FL}+\left(C_{mXF\_FR}+C_{mXB\_FR}\right)\Phi_{Z\_FR}+\left(C_{mXF\_BL}+C_{mXB\_BL}\right)\Phi_{Z\_BL}+\left(C_{mXF\_BR}+C_{mXB\_BR}\right)\Phi_{Z\_BR}$$

where *C_ZF_FL_* is the calibration coefficient obtained from the *F_ZF_* load (vertical load component applied at the front of the lower platform) and *F_Z_FL_* is the strain gauge signal calibration relationship. The other calibration coefficients were obtained in a similar way.

Lateral load *F_Y_* was measured using the upper platform signals *Φ_Y_FL_*, *Φ_Y_FR_*, *Φ_Y_BL_*, and *Φ_Y_BR_*. as described in Equation (4):

(4) $$F_{Y}=C_{Y\_FL}\Phi_{Y\_FL}+C_{Y\_FR}\Phi_{Y\_FR}+C_{Y\_BL}\Phi_{Y\_BL}+C_{Y\_BR}\Phi_{Y\_BR}$$

### 2.2. Calibration

Calibration tests were performed in the laboratory to determine the relationship between the applied loads and recorded values given by the strain gauges on both the left and right force platform. Five test configurations were performed to determine the calibration coefficient *C* for *F_Z_*, *F_Y_,* and *M_X_*. Each test was performed three times, with continuous increase of the load to reach the nominal value, both on the front and back part of the force plate. 

**Figure 3:**
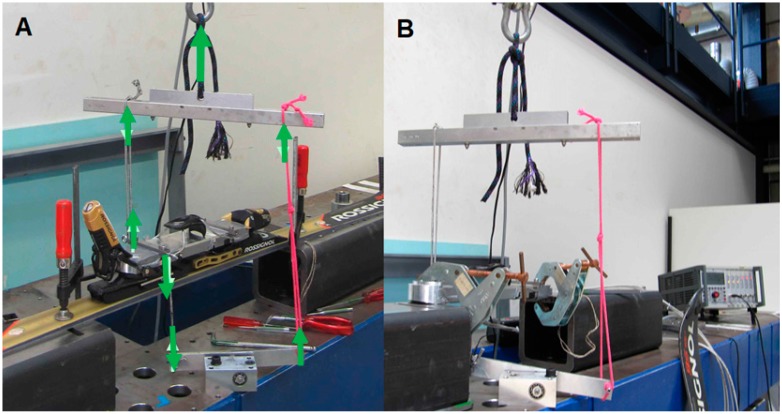
Setup used to calibrate torque around the X axis. The load was applied in pulling up the horizontal iron bar. On the left side of the platform, the load was directly applied in the upper direction, while on the right hand side, the load transited through a mechanical system that transferred the load in the top–down direction. (**A**) Setup with the ski. Green arrows indicate the load transmission. (**B**) Same setup but without the ski.

**Figure 4:**
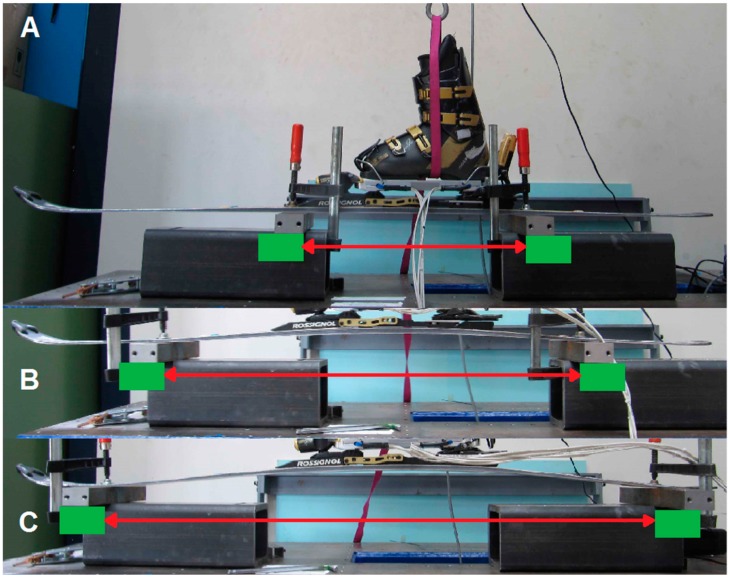
The three different fastening situations for the fifth test condition. The ski could bend between the two fastening elements (in green): (**A**) Distance of 0.5 m. (**B**) 0.9 m. (**C**) 1.3 m.

For the first configuration, the force platform was introduced in the binding, and the loads and torques applied as a usual force platform calibration process (Figure 3A) [14] for each measured component. For the second configuration, the force plate was directly attached on the calibration bench and only *Mx* was tested (Figure 3B). The next configurations concentrated on *Fz* assessment. For the third configuration, off-centered vertical loads (both positive and negative) were applied, the force plate was directly attached on the bench. For the fourth configuration, both positive and negative loads were applied on the force plate. For the fifth configuration, a vertical load was applied on the force platform with a complete setup: ski boot, force plate, and bindings mounted on the ski, using three different distances to attach the ski to the calibration bench (Figure 4A–C). Table 1 summarizes the different conditions of the calibration.

For each test, the coefficient of calibration was defined as the tension measured on the sensor divided by the applied load. Average coefficients $\tilde{C}$ were then calculated for each component and both force platforms as follow:

(5) $${\tilde{C}}_{Y}=\frac{1}{4}\left(C_{Y\_FL}+C_{Y\_FR}+C_{Y\_BL}+C_{Y\_BR}\right)$$

(6) $${\tilde{C}}_{Z}=\frac{1}{8}\left(C_{ZF\_FL}+C_{ZB\_FL}+C_{ZF\_FR}+C_{ZB\_FR}+C_{ZF\_BL}+C_{ZB\_BL}+C_{ZF\_BR}+C_{ZB\_BR}\right)$$

(7) $${\tilde{C}}_{mX}=\frac{1}{8}\left(C_{mXF\_FL}+C_{mXB\_FL}+C_{mXF\_FR}+C_{mXB\_FR}+C_{mXF\_BL}+C_{mXB\_BL}+C_{mXF\_BR}+C_{mXB\_BR}\right)$$

The following results are given for the left force platform. Coefficients of variation (the standard deviation of the calibration coefficient divided by the average calibration coefficient) were used to determine the accuracy of the different measured axis.

### 2.3. Field Test

Three European Cup racers (mean ± *SD*: total weight with equipment 116.9 ± 6.5 kg, height 1.82 m ± 0.07) participated in the field test. All participants were healthy males without any joint motion problems. The study was conducted according to the 1964 Declaration of Helsinki and written informed consent was obtained from each subject prior to participation in the study. In parallel, the study was approved by the local ethics committee of “Canton de Vaud” (number 189/11).

**Figure 5:**
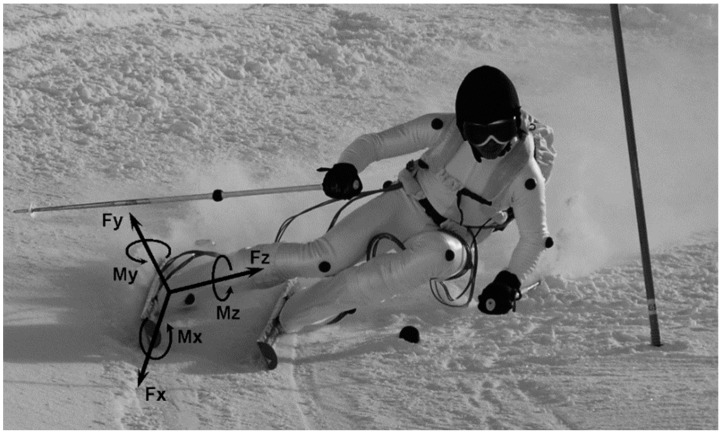
An athlete in the giant slalom equipped with the platforms and the backpack. The referential of the right force platform is also represented.

Each participant was asked to perform three runs in a giant slalom composed of six gates set up with a linear gate distance of 24 m, and a lateral offset of 9 m. The slope’s inclination angle was approximately 22 degrees. During the first three gates, the athletes increased and stabilized their speed. Data for the analysis were recorded during gates four and five. The last gate was placed to keep the rhythm. Data were recorded for the last two runs, with a frequency of 500 Hz in a datalogger placed in a backpack (Figure 5). 

The raw data obtained were filtered using a moving average on a five points window. For each axis of measure and each participant, the mean and standard deviation were calculated. Participant’s averages during a turn cycle were proposed in [N/kg] to allow for a comparison of athletes of different weights. The 95% limit of agreement (±1.96 *SD*) was also plotted to show the disparity between athletes. The evolution of the load distribution between the outside and the inside skis was also represented for a turn cycle.

## 3. Results

### 3.1. Calibration

The error on the *F_Y_* and *M_Z_* calibration was 7.3% of the applied load, 12.8% for *F_Z_* and *M_Y_*, and 1.3% for *M_X_*. Details and absolutes values can be found in Table 1.

### 3.2. Field Test

**Figure 6:**
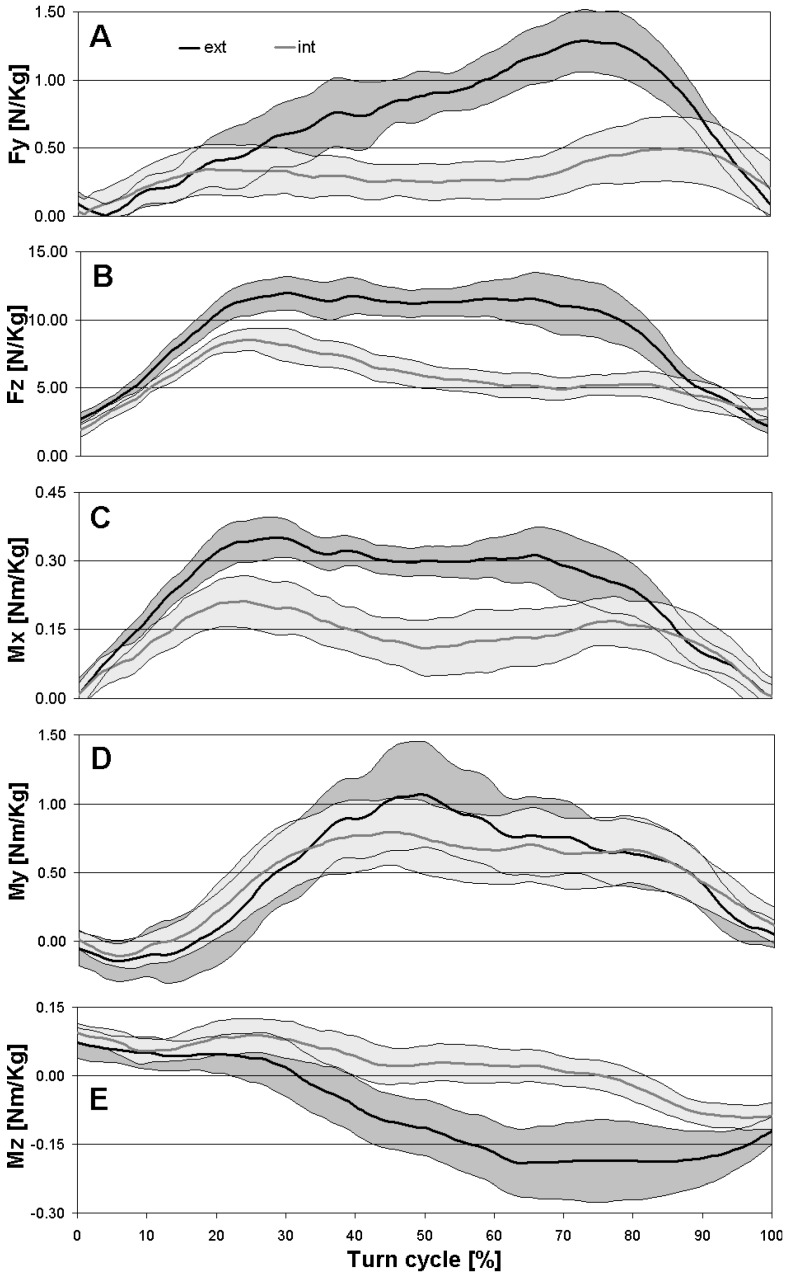
Mean Fy (**A**), Fz (**B**), Mx (**C**), My (**D**), and Mz (**E**) of three skiers and two runs for both skis during a turn cycle, with the 95% limit of agreement (grey area).

Data from the first run of each participant were removed due to the inconsistent results obtained when compared to the other two runs. Figure 6 illustrates the average results obtained with the two platforms for both the inside and outside skis. For *F_Y_*, an average standard deviation within the athletes’ runs of 19.20 N was calculated, which represents 13% of variation compared to the maximum load. The calculated *SD* was 109.11 N, or 9% of variation for *F_Z_*, 46.13 Nm (12%) for *Mx*, 202.21 Nm (13%) for *M_Y_*, and 57.50 Nm (19%) for *M_Z_*. Looking at the best repeatability between two performed runs of an athlete, an *SD* of 6% for *Fy* and *Fz* were obtained, 8% fox *Mx*, 11% for *My*, and 13% for *Mz*.

**Figure 7:**
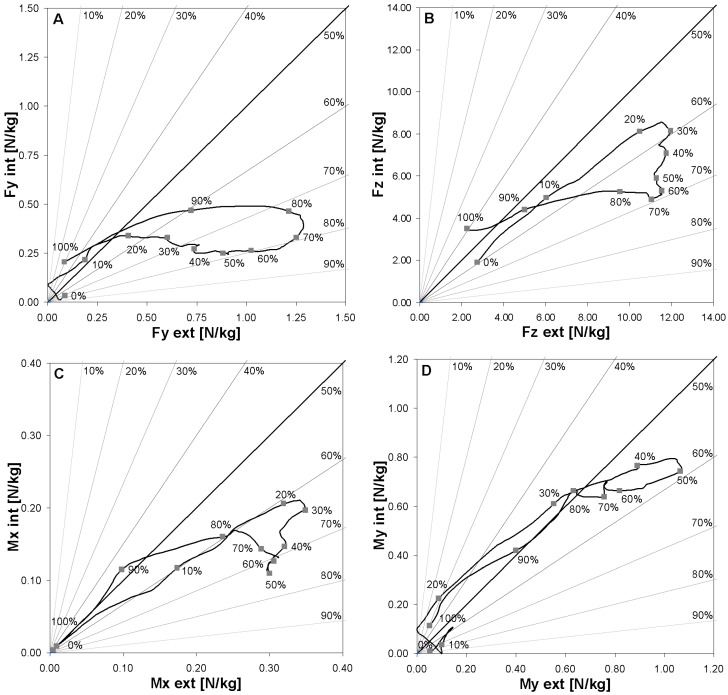
Mean Fy (**A**), Fz (**B**) Mx (**C**), and My (**D**) load distribution between the external and internal ski along the turn cycle of three skiers and two runs for both skis during a turn cycle.

To highlight the load distribution between the skis, Figure 7 plots the force distribution on the external versus the internal ski as well as the torques along the turn cycle. The 50% line represents an equal distribution. Parts of the graph on the right of that line indicate a higher proportion of load on the external ski, while parts on the left indicate higher load on the internal ski. *F_Y_* in Figure 7A indicates a distribution of about 80% on the outside ski from 50% to 70% of the turn cycle. The maximal sum of *F_Y_* int was attained around 80% of the turn cycle. *F_Z_* in Figure 7B shows an upper limit of the external ski load at approximately 1.20 N/kg. The limit was attained at 30% and maintained until 70% of the turn cycle. Regarding *M_X_*, Figure 7C offers a pattern similar to *F_Z_*, while *M_Y_* in Figure 7D indicates a very balanced distribution of the fore–aft torque between skis. 

The graph representing the load distribution obtained for *F_Y_* is different from *F_Z_* (Figure 7A,B, respectively). *F_Y_* shows a progressive increase of the forces on the outside ski while the load on the inside one remains constant. On the contrary, *F_Z_* quickly reached a maximal load on both skis, followed by a decrease of the forces on the inside ski while the load on the outside ski remained constant until the next turn transition. Fore–aft movements, illustrated by *M_Y_*, indicated similar torques on both skis, with the skier leaning backwards during the turn transition and forward during the first steering phase.

## 4. Discussion

The main result of this study is the good accuracy obtained for the calibration of the different components. The low percentage of error of the dynamometer measurements compared to the applied loads reflects a good linear behavior of each measured component. The accuracy of the force plate is slightly lower compared to the results obtained by Stricker et al. [14], but the different tests performed in the calibration process are more representative of the conditions encountered on the field. Moreover, results obtained during the field tests indicated low variability between athletes as seen by a *SD* ranging from 9% to 19%. This total variability measured includes both the variability inherent to the measurement system as well as the disparity between athletes. Looking at the best individual SD comparing two runs of the same athlete, results as low as 6% of the maximal load on the Fy and Fz components were obtained. These values show the good reproducibility of the system measurement as well as the very good and homogenous level of the skiers. Indeed, it has been demonstrated that skiers with higher skills are more capable of reproducing the exact same motion patterns than intermediate skiers [18].

The graphs obtained with the force platform are coherent with the results obtained by Klous [11] and Lüthi et al. [13]. Most of the total forces acting between the skier and the skis were measured on *F_Z_* and a higher load was measured on the outside ski. Vodickova [10] obtained similar results for *M_X_*, with higher torque on the outside ski. The amplitude and distribution of *F_Y_* found in this study are similar to the results obtained by Lüthi et al. [13].

Practice showed that when a ski boot is inserted into a binding, the boot needs to find the best adjustment. This process is done automatically during the first turns when a skier goes down the slope, and this process was imitated in the calibration process in performing different loads before starting the calibration process. During field testing, a first familiarization run was performed to allow for the correct adjustment of the force platform in the binding. Athletes reported not feeling any differences in steering the skis, indicating a good behavior of the sensor.

Benefits of using an integrated solution with strain gauges are multiple: first, sensors using strain gauges mounted in Wheatstone Bridge provide automatic compensation of temperature [19,20], which offers wide range and simplicity of use of the system. Moreover, compared to force platforms based on piezo electric sensors such as those proposed by Stricker et al. [14], signals from strain gauges do not drift, so there is no need to zero the force platform before and after each trial. A current limitation of the system is the link between static calibration and the dynamic behavior on the field. A next step to improve the accuracy of the dynamometer would be to characterize the complete ski–binding–sensor–boots–skier system from a dynamic point of view. This will allow for the eigen-frequencies of the whole system to be determined, the precise bandwidth of the sensor, and provide more detailed inputs for another design iteration.

Other sensors have been used to provide data on forces sustained by alpine skiers without any important modification of the skiers’ equipment. Nakazato et al. [21] compared a pressure insole inserted in the ski boot with a portable force platform fixed between the ski and the binding. They found that differences between the two systems depended on the phase of the turn, the skier’s level, and the type of turns performed. Gilgien et al. [22] used differential global navigation system (*DGNSS*) to estimate the external forces. This system allows for a good overall estimation of the resulting force, but cannot differentiate between the left and right leg components. Moreover, the low acquisition frequency and the antenna placement on the head of the skier does not allow for high frequency components to be determined. Inertial measurement units (*IMUs*) have also been used to determine the dynamics of ski jumping [23,24], but are restricted to a two-dimensional analysis and cannot differentiate between the constraints on the left and right legs, which is extremely important in alpine skiing.

In the future, the developed system could be used with kinematic measurements to allow quantifying loads acting on the knee as proposed by Klous et al. [11], but using the skiers’ own equipment. Finally, the system could also be combined with *GNSS* and *IMUs* [25] to determine the absolute position and orientation of the force platform, providing a fully integrated force platform.

## Figures and Tables

**Table 1:** Description of the five different tests conditions with the corresponding averaged calibration coefficients $\tilde{C}$ as described in Equations (5)–(7).

Test	Ski	Condition	Loading Position	${\tilde{C}}_{Y}$	${\tilde{C}}_{Z}$	${\tilde{C}}_{MX}$
				[kg/V]	[kg/V]	[Nm/V]
**1**	**With ski**	**Standard**	Front	0.46	1.57	0.73
			Back	0.51	1.56	0.75
2	Without ski	Standard	Front	-	-	0.74
			Back	-	-	0.73
3	Without ski	Loads applied with offset	Front	-	1.16	-
			Back	-	1.38	-
			Front	-	1.3	-
			Back	-	1.11	-
			Front	-	1.32	-
			Back	-	1.24	-
4	Without ski	Positive and negative loads	Front	-	1.34	-
			Back	-	1.34	-
5	With ski and	0.5 m attachment distance	Centered	-	1.23	-
	With boot	0.9 m attachment distance	Centered	-	1.1	-
		1.3 m attachment distance	Centered	-	1.03	-
	**Mean**			0.49	1.28	0.74
	**Standard Deviation (*SD*)**			0.04	0.21	0.01
